# Societal preference values for advanced melanoma health states in the United Kingdom and Australia

**DOI:** 10.1038/sj.bjc.6605187

**Published:** 2009-07-14

**Authors:** K M Beusterien, S M Szabo, S Kotapati, J Mukherjee, A Hoos, P Hersey, M R Middleton, A R Levy

**Affiliations:** 1 Oxford Outcomes Inc., 7315 Wisconsin Ave, 250W, Bethesda, MD 20814, USA; 2 Oxford Outcomes Ltd., 450-688 W Hastings St, Vancouver, BC, Canada V6B1P1; 3 Bristol-Myers Squibb Co., 5 Research Parkway, Wallingford, CN 06492, USA; 4 Room 443 David Maddison Building, Corner King And Watt Sts, Newcastle, NSW 2300, Australia; 5 Cancer Research UK Medical Oncology Unit, Churchill Hospital, Oxford OX3 7LJ, UK; 6 Department of Health Care & Epidemiology, University of British Columbia, Vancouver, BC, Canada V6T 1Z4

**Keywords:** melanoma, utility, standard gamble, preferences

## Abstract

**Background:**

No studies measure preference-based utilities in advanced melanoma that capture both intended clinical response and unintended toxicities associated with treatment.

**Methods:**

Using standard gamble, utilities were elicited from 140 respondents in the United Kingdom and Australia for 13 health states.

**Results:**

Preferences decreased with reduced treatment responsiveness and with increasing toxicity.

**Conclusions:**

These general population utilities can be incorporated into treatment-specific cost-effectiveness evaluations.

Advanced metastatic melanoma is almost uniformly fatal, with median survival ranging from 6 to 8 months (Korn *et al*, 2008). Therapy aims at maximising symptom control, minimising toxicity, and optimising the quality of life (Kiebert *et al*, 2003). Health status utility assessments enable the quantification of preferences for health outcomes and the estimation of quality-adjusted life expectancy. Utility measurement in melanoma to date has primarily focused on toxicities associated with interferon (IFN) therapy (Kilbridge *et al*, 2001; Dixon *et al*. 2006). No studies have obtained utilities for a broader set of health states that capture both the intended (clinical response) and unintended (toxicity) effects of available treatments for advanced melanoma. Clinical response may specifically have a substantial impact on preferences. For example, high levels of treatment-specific optimism have been associated with less depression at both the beginning and end of treatment for metastatic melanoma (Cohen *et al*, 2001). The purpose of this study was to measure preferences for a universal set of standardised health states that include clinical response and toxicities during treatment of advanced melanoma.

## Methods

A cross-sectional study was conducted in the United Kingdom and Australia to elicit utilities for advanced melanoma health states among, as recommended by the UK National Institute for Clinical Excellence, members of the general public (NICE, 2004). Trained interviewers used the standard gamble technique, which involves making decisions under conditions of uncertainty (Torrance, 1986). Respondents imagine that they are in a selected health state. They can remain in that state, or take a gamble that involves a chance (*p*) of achieving full health with a corresponding chance (1−*p*) of being dead. The *p* probabilities are varied using a ping-pong approach, converging on *p*=50%, until the respondent is indifferent between the two options. For each health state, the respective utility equals the probability *p* of full health at the point at which the respondent is indifferent between remaining in the health state and taking the gamble. Utility scores range from 0.0, reflecting being dead, to 1.0, reflecting full health.

Respondents were recruited from the United Kingdom and Australia in December 2007. All participants provided informed consent and received compensation for their time. This study was approved by the Independent Investigational Review Board (Plantation, FL, USA) and complied with the tenets of the Declaration of Helsinki.

### Health state development

Four melanoma treatment-related response states, one symptomatic melanoma state, and nine toxicity-related health states were developed on the basis of published literature. Specifically, treatment response status was described on the basis of the World Health Organisation's definition for all cancers (WHO, 1979). Partial response state was based on a >50% decrease in lesion mass; stable disease was based on a <25% decrease or increase in lesion mass; and progressive disease (PD) was based on the appearance of new lesions or increase by >25% in lesion mass. In addition, a best supportive care (BSC) state represented no indicated or desired cancer treatment, and a symptomatic melanoma health state represented symptoms experienced in advanced melanoma.

Toxicity health states were selected on the basis of common grade I/II toxicities (occurring in ⩾10% of treated patients) from published and unpublished literature, and product inserts for ipilimumab, dacarbazine, temozolomide, interleukin-2, fotemustine, and IFN-*α*. Grade III/IV toxicities were described in two health states: one involving outpatient treatment for 1–2 days and the other involving hospitalisation for 2–5 days. Toxicity health state descriptions were developed using the Common Terminology Criteria for Adverse Events (Cancer Therapy Evaluation Program, 2006). One melanoma treatment, ipilimumab, may be associated with immune-related adverse events that may lead to therapeutic benefit (Downey *et al*, 2007; O'Day *et al*, 2007). Thus, for exploratory purposes, we included a health state for partial response plus toxicity as indication that treatment is working.

The health states were described as being treated for cancer (melanoma was not specified), whether or not treatment is working, and changes in tumour size, pain levels, appetite, effort required to perform daily activities, and fatigue. Each of the toxicity descriptions was described in association with partial response so that the respective utility decrements for toxicities could be calculated by subtracting the utility for partial response from the utility of the toxicity state. All health states were labelled with symbols to avoid imposing a predetermined hierarchical order on the states. The descriptions were developed in layperson terms, and health states were refined after an iterative review by five clinical experts, two oncology nurses, three quality-of-life researchers, and a pilot test with individuals from the general public.

## Results

There were a total of 140 participants, 77 from Australia and 63 from the United Kingdom, included in the analysis. The mean respondent age was 45±14 years, and 48% were male. The samples were well matched according to the demographic distributions of their target adult populations (England and Wales Census, 2001/2007; Australian Bureau of Statistics, 2007a, 2007b).

Table 1 reports the mean utilities for the health states. Partial response was most preferred, followed by stable disease, and PD and BSC were equivalently valued within countries. A comparison between countries revealed higher mean utilities in Australia than in the United Kingdom for partial response (0.91 *vs* 0.85; *P*<0.001) and stable disease (0.83 *vs* 0.77; *P*=0.018). However, mean utilities were lower among Australians compared with those from the United Kingdom for the least favoured health states of PD (0.47 *vs* 0.59; *P*=0.001) or BSC (0.46 *vs* 0.59; *P*<0.001). Mean utilities did not differ by age or sex.

The higher mean utility for partial response observed in Australia relative to the United Kingdom translated into toxicities being valued less favourably by Australian respondents when considered as toxicity-specific utility decrements. (These decrements can be added to the utilities for each of the base states (CR, PR, SD, and PD), as applicable.) Hair loss had the smallest utility decrement in both countries (–0.03) and hospitalisation for grade III*/*IV toxicity had the greatest utility decrement (−0.13 (United Kingdom) *vs* −0.20 (Australia)). Symptomatic melanoma was also the least preferred, with values similar to hospitalisation for grade II/IV toxicity. The exploratory health state, toxicity as an indication that treatment is working, was associated with similar decrements between the countries of Australia (−0.08) and the United Kingdom (−0.09) (data not shown).

## Discussion

This study yielded general population utilities for a universal set of advanced melanoma health states. These utilities can assist in the evaluation of melanoma treatments in future studies. As may be expected, preferences for health states decreased with reduced treatment responsiveness and with an increasing grade of treatment-related toxicity.

The development of health state vignettes made it possible to gauge the impact of knowledge of treatment response on individual preferences: the mean utility for partial response was higher than that for stable disease by 0.08 in both Australia and the United Kingdom. In addition, the utility decrement for the exploratory health state describing severe toxicity that may be indicative of the treatment working was approximately twice as favourable, relative to the other severe grade III/IV toxicity states (−0.08 *vs* −0.13 and −0.17). Using a vignette approach allowing for descriptions of clinical response *vs* a generic utility questionnaire such as the EQ-5D can lead to different estimates of comparative effectiveness. Specifically, decreases in quality-of-life during IFN-*α* treatment for advanced melanoma were offset by reduced risk of recurrence and mortality when vignette-based utilities were applied (Kilbridge *et al*, 2002; Crott, 2004; Crott *et al*, 2004). In contrast, IFN was only marginally better in treating melanoma than was BSC when generic EQ-5D utilities were used (Dixon *et al*, 2006).

More research is needed on the impact of prognosis on treatment-related utilities. Hope is a powerful influence and a key factor in finding the motivation to continue being positive about life (Cohen *et al*, 2001). Therefore, it is not too surprising that knowing that treatment is working translates into a high utility. However, it is unknown how different lengths of perceived survival impact a patient's hope and preferences. Richardson *et al* (1996) found that utilities for a multi-phase health scenario covering 16 years were not commensurate with conventionally calculated quality-adjusted life years, the sum of the years in each health state multiplied by the utility for each state. They found that knowledge of future suffering and death casts a shadow over (or devalues) enjoyment of the earlier years. However, as the researchers discuss, patients would normally be provided with information on possible prognosis and not given a scenario wherein outcome is deterministic. In this regard, the approach used in our study may be preferable, given that prognosis is a key influential component in the assignment of health state utilities.

We observed significant differences in mean health state utilities between countries for the clinical response states. Relative to UK respondents, Australians reported a lower impact of the less-severe clinical response states and a greater impact of the more-severe response states. Similarly, studies using indirect methods, such as the EQ-5D, to estimate patient preferences have shown quite large inter-country differences, including in patients with cancer (Sullivan *et al*, 2005; Pickard *et al*, 2007). The higher mean utility for partial response in Australia relative to that in the United Kingdom translated into toxicities being valued less favourably by Australian respondents when considered as toxicity-specific utility decrements.

It is unknown whether the magnitude of utility decrements for toxicities would be influenced by using stable disease as the base state instead of partial response. Moreover, coupling toxicities with a disease state limits the opportunity to measure the true contribution of each component to the resulting utilities. However, combining these various impacts more truly reflects reality. For future applications, these decrements can be coupled with the utility of any respective clinical response, as applicable. However, analysts should be cautious when applying the utility estimates from this study, keeping in mind that the differences observed in toxicity state utilities were driven by the different utilities assigned to the base case of partial response.

This study did not include a health state for complete response. If included, respondents would be required to imagine that they had melanoma but that it was in complete remission; we felt that this may be too complex an undertaking to include as a part of the standard gamble exercise. However, an estimate for living disease free has been reported by Kilbridge as 0.96 (Kilbridge *et al*, 2001). In addition, our study did not consider health states with multiple toxicities. Several recent studies have explored the estimation of utilities given this scenario. Dale *et al* (2002) and Fu and Kattan (2008) recommend using a minimum model, which predicts a joint-state utility as being equal to the lower of the two given single-state utilities for an individual. However, Flanagan *et al*. (2006) recommend the use of a multiplicative model. Further research is needed in this area.

The study reports general population health state utilities from the United Kingdom and Australia for a universal set of advanced melanoma health states, including potential clinical responses and toxicities associated with various treatments. This study used a rigorous process for the development of standardised health states that incorporated both intended treatment responses and unintended events. The utilities generated in this study can be applied in future cost-effectiveness analyses of treatment for advanced melanoma, as well as those for the treatment of potentially other types of advanced cancer with similar prognoses.

## Figures and Tables

**Table 1:** Mean utilities of base states and utility decrements associated with treatment toxicities

**Health state**	**All mean (s.e.)**	**Australia mean (s.e.)**	**UK mean (s.e.)**
*Clinical response states*			
Partial response	0.88 (0.01)	0.91 (0.01)	0.85 (0.02)
Stable disease	0.80 (0.01)	0.83 (0.01)	0.77 (0.02)
Progressive disease	0.52 (0.02)	0.47 (0.03)	0.59 (0.02)
Best supportive care	0.52 (0.02)	0.46 (0.03)	0.59 (0.02)
			
*Utility decrement for toxicity states*			
Hair loss (grade I/II)	−0.03 (0.01)	−0.03 (0.01)	−0.03 (0.01)
Skin reaction (grade I/II)	−0.06 (0.01)	−0.08 (0.01)	−0.03 (0.01)
Diarrhoea (grade I/II)	−0.09 (0.01)	−0.11 (0.01)	−0.06 (0.01)
Nausea/vomiting (grade I/II)	−0.10 (0.01)	−0.12 (0.01)	−0.07 (0.01)
Flu-like syndrome (grade I/II)	−0.11 (0.01)	−0.13 (0.01)	−0.09 (0.01)
Stomatitis (grade I/II)	−0.13 (0.01)	−0.14 (0.01)	−0.10 (0.02)
1-day in-/outpatient stay for severe toxicity (grade III/IV)	−0.13 (0.01)	−0.14 (0.01)	−0.11 (0.02)
Symptomatic melanoma^a^	−0.16 (0.01)	−0.20 (0.02)	−0.11 (0.02)
2–5-day hospitalisation for severe toxicity (grade III/IV)	−0.17 (0.01)	−0.20 (0.02)	−0.13 (0.02)

a Not a treatment-related toxicity; describes symptoms associated with progressive melanoma.
